# Editorial: Streaming Inflammation: From Damage to Healing and Resilience

**DOI:** 10.3389/fphar.2022.969453

**Published:** 2022-07-12

**Authors:** Pallavi R. Devchand, Eric E. Schadt, Garret A. FitzGerald

**Affiliations:** ^1^ Department of Physiology and Pharmacology, University of Calgary, Calgary, AB, Canada; ^2^ SEMA4, Stamford, CT, United States; ^3^ Department of Systems Pharmacology and Translational Therapeutics, Perelman School of Medicine, University of Pennsylvania, Philadelphia, PA, United States

**Keywords:** resilience, disease states, drug target, healing, identity, trafficking

“ We were born before the wind

Also younger than the sun... ”

-*Van Morrison* in *Into the Mystic (1970*).

Every human is a multiplex of ecosystems. The balance of symbiosis and competition from within is as critical as maintenance and defense systems, like inflammation. To achieve homeostasis, these adaptive processes occur at different stages in different scenarios - all over the body - at the same time, and often unnoticed. How are the streams of molecular and cellular players coordinated in systemic responses that maintain integrity of the whole? This collection of articles aims to spotlight three dynamic relationships: damage, healing and resilience.

In a pilot study on Sjogren’s Syndrome, Das et al. profile human saliva and tears using a proteomics workflow to create a proof-of-concept working model for examining altered protease activity associated with the damage inflicted in this rare disease.

Three articles target catabolism of bioactive lipids and impact on responsiveness to high fat diet. In LPS-induced human phagocytes, Tang et el didactically examine leukotriene biosynthesis pathways to arrive at a fitting model of the ATP-binding domain of p38 to KIRA6, a widely-used inhibitor of the IRE1alpha component of the unfolding protein response with p38. Using knock-out mice of soluble epoxide hydrolase, Wagner et al. associated metabolism of high fat diet and diet-induced obesity to organ-selective observations of sex-specific responsiveness. Along this same vein, Ulu et al. opted to modulate endogenous polyunsaturated fatty acid levels by using a fatty-acyl transferase transgenic mouse, and evaluated impact on lung inflammation triggered by repetitive exposure to liquid dust.

In the healing process, Cen et al. focused on lipopolysaccharide-induced migration and proliferation of human dermal fibroblasts to identify a role for strathmin via p38 mitogen-activated protein kinase. With a novel twist of local scar formation and liver function, Lyu et al. measured effects of combinatorial drug treatments (anisodamine and neostigmine) on local and chronic inflammation in a rat model of biliary obstruction.

Several articles argue for fresh perspective in dynamic disease scenarios. After framing recent advances in the complex and fluid roles of macrophages in different stages of atherosclerosis pathogenesis, Farahi et al. propose provocative macrophage-based therapies for disease management. From an alternate perspective on atherosclerosis, Botts et al. focus on vascular endothelial cells as drivers of disease, and postulate on molecular approaches to re-routing inflammation away from disease progression. Ballerini et al. weave us through chronic inflammation in select cancers and liver diseases to hone in on the roles of platelets, and the potential of platelets as target cell types for biomarkers and drug therapy. In their white paper, Zhu et al. take a step out of the fray of activated immune traffic and argue for the relevance of dynamics in the ecosystems of somatic cells, particularly in the subtle context of personalized health and medicine over a lifetime.

**FIGURE 1:**
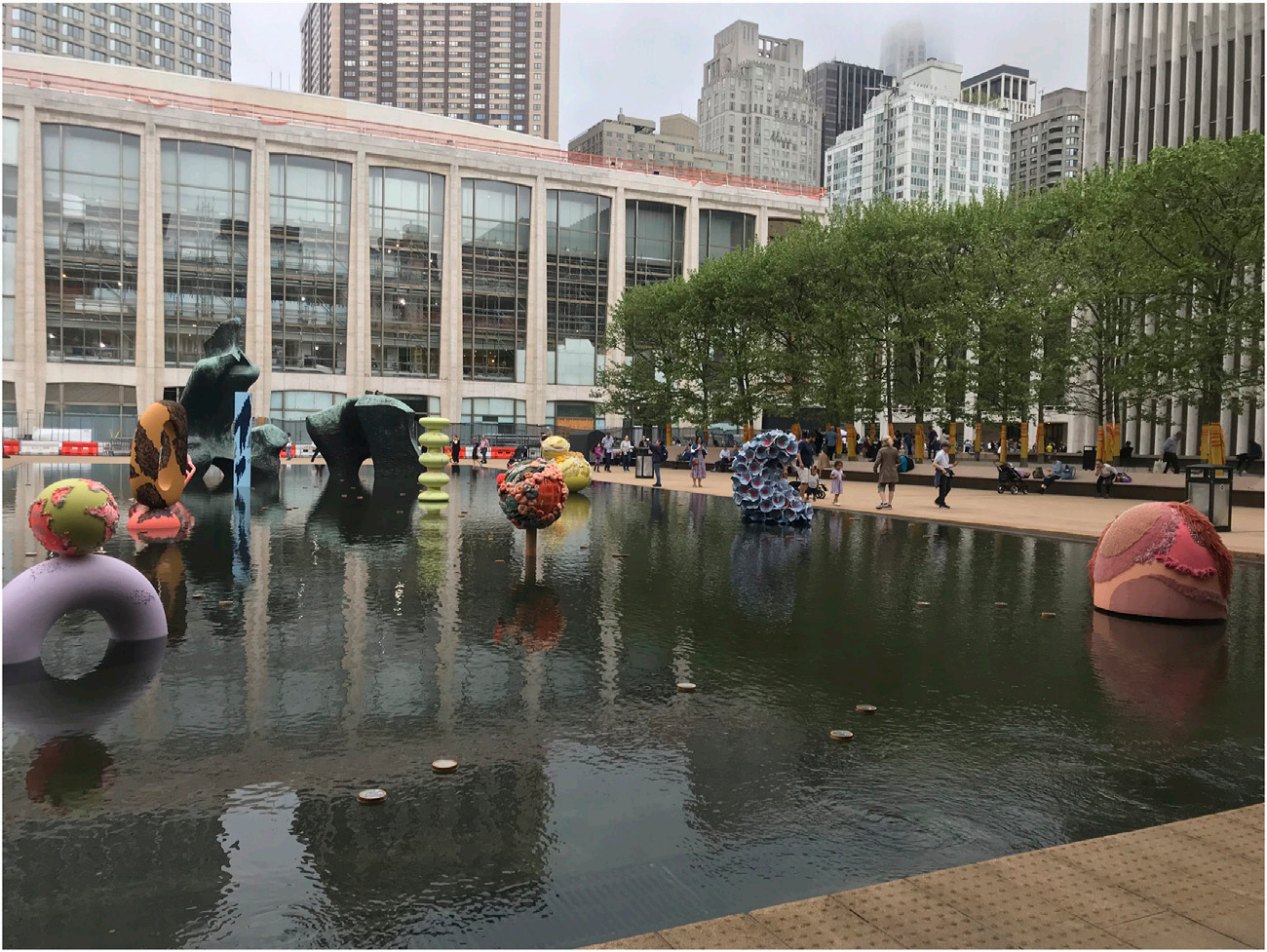
Islands in The Sea. A series of Monuments and Rituals by Amanda Phingbodhipakkiya at the Paul Milstein Reflecting Pool, New York. Photo by Pallavi R. Devchand.

On earth, Devchand noted the scientific and artistic drivers of human complexity in personal expressions of resilience, susceptibility and passion (Figure 1). And out of this world, the brave Millie Elizabeth Hughes-Fulford (1945–2021) lived her mantra of an astronaut-scientist:

“To dream and to explore is the nature of humankind.

And to find new knowledge is key.”

